# Amniotic Membrane Transplantation for Wound Healing, Tissue Regeneration and Immune Modulation

**DOI:** 10.1007/s12015-025-10892-x

**Published:** 2025-05-14

**Authors:** Uday Pratap Singh Parmar, Pier Luigi Surico, Anna Scarabosio, Vincenzo Barone, Rohan Bir Singh, Fabrizio D’Ancona, Marco Zeppieri, Pier Camillo Parodi, Tommaso Mori, Francesco Cutrupi, David Hui-Kang Ma, Antonio Di Zazzo, Marco Coassin

**Affiliations:** 1 Department of Ophthalmology, Government Medical College and Hospital, Chandigarh, 160030 India; 2 Department of Ophthalmology, Campus Bio-Medico University Hospital, Rome, 00128 Italy; 3 Department of Organs of Sense, University of Rome La Sapienza, Rome, 00185 Italy; 4 Department of Plastic Surgery, University Hospital of Udine, Udine, 33100 Italy; 5 Discipline of Ophthalmology and Visual Science, Faculty of Health and Medical Sciences, Adelaide Medical School, University of Adelaide, Adelaide, Australia; 6 Department of Ophthalmology, Royal Liverpool University Hospital, Liverpool, UK; 7 Department of Ophthalmology, University Hospital of Udine, Udine, 33100 Italy; 8 Department of Ophthalmology, Chang Gung Memorial Hospital, Linkou, Taiwan; 9 Department of Ophthalmology, Xiamen Chang Gung Hospital, Xiamen, China; 10 Department of Chinese Medicine, College of Medicine, Chang Gung University, Taoyuan, Taiwan; 11 Center for Tissue Engineering, Chang Gung Memorial Hospital, Linkou, Taiwan

**Keywords:** Amniotic membrane, Transplantation, Stem cells, Wound healing, Immunology, Tissue regeneration, Ophthalmology, Nanoparticles, Gene therapy, Nerve regeneration

## Abstract

Amniotic membrane transplantation (AMT) has emerged as a versatile therapeutic modality with significant applications in wound healing, tissue regeneration, and ophthalmology. This review comprehensively evaluates AMT's efficacy in acute and chronic wound management, where it has been shown to alleviate pain, reduce infection risk, and facilitate epithelialization. In chronic wounds, AMT enhances healing through mechanisms such as re-epithelialization, angiogenesis, and immune modulation. Additionally, AMT exhibits promise in nerve regeneration, demonstrating potential in the repair of peripheral and central nervous system injuries by fostering neural recovery and minimizing scar formation. In ophthalmology, AMT is instrumental in corneal surface reconstruction, conjunctival repairs, and the management of dry eye syndrome and limbal stem cell deficiency. While the benefits of AMT are well-documented, this review also addresses significant challenges, including variability in success rates across different clinical conditions, ethical concerns regarding donor tissue usage, and regulatory hurdles impacting its broader clinical application. Furthermore, we integrate recent advances in the understanding of AMT's molecular mechanisms – such as its antioxidant effects via Nrf2/HO-1 pathway and immune modulation via P2X7 receptor pathways – and highlight innovative strategies including the incorporation of nanoceria nanoparticles, Vitamin D3 supplementation, and gene therapy approaches to enhance AMT outcomes. By exploring these dimensions, the review highlights not only the current state of AMT but also its potential future role in advancing regenerative medicine, including emerging applications in spinal cord repair, orthopaedics, and tissue engineering. This updated synthesis aims to inform clinicians and researchers about the multifaceted applications of AMT, promoting further investigation and optimization of this promising therapeutic approach.

**Graphical Abstract:**

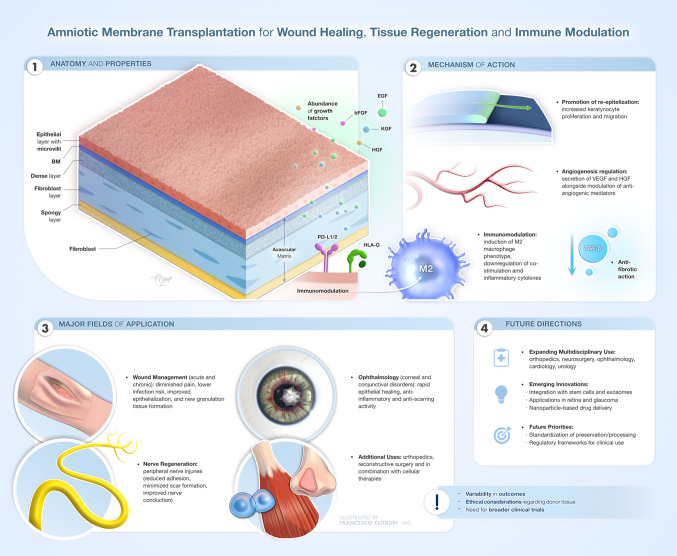

## Introduction

The amniotic membrane (AM), or amnion, is the innermost layer of the placenta that surrounds the fetus, consisting of a thin epithelial layer, a thick basement membrane, and an avascular stromal matrix [1].

Its unique biological properties have made it a valuable biomaterial in medicine for over a century. The first recorded therapeutic use of amniotic tissue dates back to 1910, when John Davis successfully used human amnion as a skin graft to treat burns and wounds [2]. In 1913, Sabella similarly used fetal membranes for skin grafting, recognizing their potential to promote wound healing [3]. Early pioneers like Stern in the 1910 s and de Rötth in 1940 expanded AM applications to surgical fields; de Rötth applied fetal membranes in ophthalmology for conjunctival defect repair as a substitute for rabbit peritoneum in severe chemical burns [4]. These initial attempts, however, met with mixed success due to limited understanding and preservation techniques at the time. Interest in AMT was rejuvenated in the late twentieth century – notably, Kim and Tseng (1995) reintroduced preserved human amniotic membranes for ocular surface reconstruction, demonstrating successful corneal surface healing in severely damaged eyes [5]. This pivotal work sparked widespread adoption of AMT in ophthalmology and renewed scientific inquiry into amnion’s therapeutic mechanisms.

Today, AMT is used as a biological dressing and graft in numerous disciplines, ranging from dermatology and plastic surgery to orthopedics and ophthalmology. It serves as a natural scaffold that not only covers wounds but actively promotes tissue repair through its rich content of growth factors, anti-inflammatory cytokines, and extracellular matrix components.

The scope of this review is to provide a comprehensive and up-to-date overview of AMT in wound healing and tissue regeneration, integrating findings from recent literature to address its mechanisms of action, clinical applications across various fields (acute and chronic wounds, nerve and ocular tissue regeneration, etc.), and emerging innovations. We will also discuss the immunological aspects of AMT, including microchimersim and immunomodulation, which are crucial for understanding long-term outcomes. In doing so, we aim to highlight both the therapeutic successes of AMT and the challenges and future directions for research.

## Properties of Amniotic Membrane

### Anatomy and Structural Composition

The human amniotic membrane varies in thickness from 0.02 mm to 0.5 mm and is an avascular tissue comprised of multiple distinct layers. In a classic study, Gordon L. Bourne (1960) described the amnion as consisting of five layers: a single epithelial layer, a basement membrane, and a trilaminar mesenchymal stroma subdivided into a compact layer, an intermediate fibroblast layer, and an outer spongy layer [6].

The epithelial layer is composed of metabolically active cuboidal or columnar cells with apical microvilli, [7] which increase in density over gestation and are covered by an amorphous substance at term [8]. Just beneath, the basement membrane contains a network of collagen types IV and VII, elastin, laminin, fibronectin, and glycosaminoglycans (like hyaluronic acid), providing a supportive scaffold for cellular adhesion and migration. This layer also harbours matrix metalloproteinase inhibitors and various bioactive molecules (cytokines, growth factors) that contribute to wound healing [9, 10].

The stromal matrix underlying the basement membrane can be further divided into: a compact inner layer rich in collagen that provides tensile strength to the membrane; a looser intermediate layer containing fibroblasts; and an outer spongy layer (zona spongiosa) adjacent to the chorion, which contains proteoglycans and serves as a cushion and connection between amnion and chorion [6]. This unique architecture endows the amnion with elasticity, mechanical stability, and the ability to integrate into host tissues without triggering robust immune rejection, owing in part to its lack of vasculature and expression of immunogenic antigens. The amniotic basement membrane in particular acts as a biological substrate that can facilitate the re-epithelialization of wounds by supporting the growth of epithelial cells over its surface [11].

In summary, the structural composition of AM establishes the foundation for its biological functions, creating a natural scaffold that is both biocompatible and bioactive.

### Bioactive Factors and Mechanisms of Action

Beyond its structural role, the amniotic membrane is often described as a reservoir of bioactive molecules that modulate healing.

#### Growth Factors

The growth factors abundantly present in or released by AM play a pivotal role in tissue regeneration. Koizumi et al. (2000) demonstrated that preserved human amniotic membrane expresses mRNA and proteins for multiple growth factors – including hepatocyte growth factor (HGF), keratinocyte growth factor (KGF), epidermal growth factor (EGF), and basic fibroblast growth factor (bFGF) – especially when the amnion’s epithelium is intact [12]. The epithelial cells of AM appear to be a major source of these factors, which can stimulate the proliferation and migration of adjacent epithelial cells in a wounded area. For instance, amniotic membrane homogenate (AMH) containing EGF, bFGF, interleukin-6 (IL-6), and IL-8 has been investigated as a topical therapy for refractory corneal epithelial defects, demonstrating measurable concentrations of these cytokines and growth factors and suggesting efficacy in promoting epithelial healing [13].

Furthermore, modifications of the AM to enhance its growth factor content have shown promise; one study reported that heparinized, growth-factor enriched human acellular amnion significantly accelerated wound closure in a murine model, particularly during the first week of healing [14]. Overall, AM-derived growth factors such as EGF, KGF, HGF, and bFGF are key contributors to its pro-healing effect and have been implicated in stimulating cell proliferation, enhancing epithelialization, and even promoting angiogenesis in damaged tissues.

#### Immunomodulatory Effects

In addition to growth factors, the immunomodulatory effects of the amniotic membrane are crucial to its therapeutic action. Amniotic cells (both epithelial and mesenchymal cells within the membrane) exhibit a low immunogenic profile: they express HLA class I molecules at baseline but negligible HLA class II, [15] and they upregulate the immunosuppressive non-classical HLA-G molecule. HLA-G on AM interacts with inhibitory receptors (ILT2, ILT4, KIR2DL4) on immune cells, [16] dampening immune responses – for example, HLA-G can inhibit dendritic cell maturation and promote tolerance [17]. Moreover, amniotic cells can express the checkpoint molecules PD-L1 and PD-L2, which engage PD-1 on T cells and other leukocytes to reduce inflammatory cytokine secretion (like IFN-γ, TNF-α, IL-2) and inhibit T cell proliferation [18]. Through these mechanisms, AM transplantation creates a localized immunosuppressive environment, protecting the wound from excessive inflammation that could impede healing or cause scarring [19].

Notably, soluble factors from AM influence immune cell behaviour: for example, migration inhibitory factor (MIF) secreted by amniotic epithelial cells prevents neutrophils and macrophages from infiltrating tissues, as evidenced by in vitro assays and animal models where polymorphonuclear cells adhering to AM undergo apoptosis [20]. Similarly, conditioned media from amniotic mesenchymal cells drives macrophages toward an anti-inflammatory M2 phenotype, with a marked reduction in pro-inflammatory cytokines (IL-1α, IL-1β, IL-12, IL-8, TNF-α) and an increase in anti-inflammatory IL-10 [21].

The presence of an amniotic membrane can also induce apoptosis in activated immune cells: studies in a necrotizing keratitis model showed increased lymphocyte and macrophage apoptosis when exposed to the amniotic stromal matrix [22]. These immunomodulatory properties contribute to AMT’s ability to reduce scar formation and support regenerative healing. In fact, the amniotic stroma contains factors that directly inhibit fibrosis; it has been shown to suppress TGF-β signalling in fibroblasts, thereby preventing myofibroblast differentiation and excessive scar tissue deposition [23].

Beyond classical growth factors and cytokines, emerging research has shed light on AM’s role in oxidative stress and inflammatory pathways. Amniotic membrane-derived components can activate the Nrf2/HO-1 pathway, a critical cellular defense mechanism against oxidative stress. In a hypoxia-injured cardiac cell model, pretreatment with human amniotic membrane protein extract significantly increased cell survival and reduced lactate dehydrogenase release, an effect tied to upregulation of nuclear factor erythroid 2-related factor 2 (Nrf2) and its downstream enzyme heme oxygenase-1 (HO-1) [24]. This activation led to the inhibition of the high-mobility group box-1 (HMGB1) and MyD88 inflammatory cascade, thereby protecting cells from apoptosis [24]. These findings indicate that AM can exert cytoprotective, anti-oxidative effects at the molecular level – by boosting Nrf2/HO-1, AMT may mitigate oxidative stress in wounds and ischemic tissues, contributing to improved healing outcomes.

Additionally, AM and its derivatives might influence purinergic signalling involved in inflammation. The P2X7 receptor, an ATP-gated ion channel on immune cells, is known to drive inflammasome activation and chronic inflammation [25]. While not yet extensively studied in the context of AMT, it is noteworthy that natural compounds targeting P2X7 (such as the diterpenoid dihydrotanshinone) can preserve tissue integrity in models of diabetic retinopathy by inhibiting P2X7-mediated inflammatory damage [25]. This suggests future avenues where AMT could be combined with P2X7 receptor modulation to further control inflammation in regenerative treatments. In summary, the amniotic membrane’s mechanisms of action are multifaceted – it provides a scaffold for cell growth, supplies key growth factors, and modulates the immune response and redox environment of the wound.

## Amniotic Membrane Transplantation in Wound Healing

The use of AMT in wound management is one of its most well-established applications. Amniotic membrane derivatives have been applied as biological dressings in both acute and chronic. Multiple clinical studies have demonstrated that AMT can accelerate wound closure, reduce pain and scarring, and lower infection rates, owing to the membrane’s unique combination of epithelial support, pro-angiogenic factors, and anti-inflammatory action.

### Acute Wounds

In acute wounds, AMT serves as a temporary biological covering that protects the wound and actively encourages healing. For example, Choudhary et al. studied 60 patients with large traumatic wounds and found that those treated with amniotic membrane grafts experienced significantly less exudate, reduced peri-wound edema, and lower pain scores compared to conventional care [26]. The AMT group also had fewer infections and debridements, indicating that the amnion provided an antimicrobial and wound-stabilizing benefit [26]. In the setting of burns, Meller et al. reported that AMT applied to acute ocular burns resulted in rapid epithelialization in 84.6% of eyes within 2–5 weeks, with notable visual improvement in moderate-grade burns [27]. Although AMT did not prevent limbal stem cell deficiency in the most severe grade IV burns, it clearly promoted surface healing and reduced inflammation in milder cases. Another randomized controlled trial (RCT) in severe ocular chemical injuries found that adding AMT to standard medical therapy did not significantly speed up corneal epithelial healing or final vision compared to medical therapy alone [28].

This suggests that in extreme injury, the benefits of AMT may be limited by the extent of stem cell loss or other factors; however, early application of AM (within the first-week post-burn) has been suggested to improve outcomes such as reduced corneal neovascularization and symblepharon (adhesion) formation when combined with conventional treatment [29].

In cutaneous burns, both historical and recent evidence support AMT’s efficacy. As early as the mid-twentieth century, humans with second and third-degree burns were treated with amniotic membranes to reduce pain and promote re-epithelialization, an approach that has been refined over time. Modern clinical series have used dehydrated human amnion/chorion membranes (dHACM) as skin graft alternatives [30]. In pediatric burn patients, for instance, the application of dHACM has yielded faster healing rates compared to standard autografting [30].

The biological cover provided by the amnion not only keeps the wound moist (preventing desiccation) but also delivers growth factors like EGF and bFGF directly to the wound bed, which can speed up the formation of new epithelium and dermis.

Additionally, because AM is thin and semi-translucent, clinicians can easily monitor the wound through the membrane for signs of infection or healing progress. Acute surgical wounds have also benefited: for example, amniotic membrane dressings on split-thickness skin graft donor sites significantly increased the rate of epithelialization and decreased pain compared to conventional dressings [30]. These outcomes underscore AMT’s dual role as a protective dressing and a bioactive therapy in acute wound care.

### Chronic Wounds

Chronic wounds, by definition, are wounds that fail to progress through the normal stages of healing and persist for weeks or months [31]. These include diabetic ulcers, venous leg ulcers, pressure ulcers, and other ulcers often characterized by poor perfusion, persistent inflammation, and bacterial colonization. AMT has shown particular promise in managing chronic, non-healing wounds by jump-starting the healing process through its pro-regenerative and anti-inflammatory properties [32]. Chronic wound beds typically suffer from a deficient healing environment: they often have excessive proteases that degrade growth factors, [33] reduced levels of cytokines necessary for cell migration, [33] and senescent cells that no longer respond to healing signals [31]. The amniotic membrane can help correct some of these imbalances. It provides exogenous growth factors and cytokines that may be lacking, and its presence has been noted to alter the wound biochemistry favourably – for instance, by reducing levels of proteolytic enzymes and creating a scaffold for cell migration and angiogenesis [34].

A meta-analysis by Mohammad et al. examined 11 RCTs of dehydrated amnion/chorion allografts in chronic wounds and found that AMT, when added to standard wound care, significantly improved the rate of complete wound healing and reduced healing time at 6 and 12 weeks [35]. In most trials, a greater proportion of patients achieved full closure of their ulcers with AM grafts than with conventional treatments alone​. Another systematic review by Lakmal et al. similarly concluded that the majority of high-quality studies report superior wound closure rates with amniotic membrane products compared to controls [36]. For example, in a multicenter trial on chronic venous leg ulcers, patients treated with a weekly applied dehydrated human amnion/chorion membrane (DHACM) had a 75% healing rate at 12 weeks versus 30% in those receiving compression therapy alone [37]. These results highlight the clinical efficacy of AMT in chronic wound care.

Clinically, wounds treated with AMT often show rapid granulation tissue formation and a reduction in fibrinous exudate/slough. Mermet et al. observed that in 15 patients with chronic venous ulcers, use of AM led to increased granulation and decreased fibrin on the wound surface within 2 weeks; notably, 80% of these patients had significant improvement, and several wounds that had been unresponsive for months achieved complete healing within 3 months of AMT [38].

### Mechanisms in Wound Healing

The superior performance of AMT in chronic wounds can be attributed to several mechanisms:

#### Re-Epithelialization

AMT strongly promotes re-epithelialization, which is often stalled in chronic wounds. The membrane releases EGF and transforming growth factor-β (TGF-β), among other factors, which stimulate keratinocyte migration and proliferation at the wound edges [39, 40]. In chronic ulcers, keratinocytes are frequently trapped in a hyperproliferative but non-migratory state. Amniotic membrane placement has been shown to reset this state – it provides a temporary basement membrane for keratinocytes to migrate over and supplies low levels of TGF-β along with members of the EGF family to modulate the epidermal microenvironment [41]. AM also reduces TGF-β-induced phosphorylation of SMAD2/3 in keratinocytes, thereby downregulating cell cycle inhibitors (p21 and p15) and allowing cells to re-enter the cell cycle and move across the wound [42]. Another observation in chronic wounds treated with AM is the upregulation of c-Jun in keratinocytes at the wound edge. c-Jun is a component of the AP-1 transcription factor needed for cell migration; its induction suggests that AMT helps reactivate the genetic program for wound closure that had stalled [43, 44]. Together, these effects explain why epithelial resurfacing is accelerated under the amnion.

#### Angiogenesis

Chronic wounds often lack sufficient blood vessel growth, leading to hypoxia and poor nutrient delivery in the wound bed. The amniotic membrane has angiogenic properties that counteract this issue. Yu et al. demonstrated in a hypoxic wound model that AM application significantly boosted angiogenesis, with upregulation of 25 angiogenesis-related proteins including angiogenin, VEGF, MMP-3, MMP-9, IL-1α, MCP-1, and HGF [45]. By increasing pro-angiogenic factors, AMT promoted robust microvascular network formation in the wound, thereby improving perfusion and oxygenation. Another study in rats found that amniotic membrane extract increased the expression of angiogenic markers (e.g., VEGF, CD31) in surgical wounds, correlating with better healing [46]. Interestingly, AM contains some anti-angiogenic factors as well (such as thrombospondin-1 and pigment epithelium-derived factor in the basement membrane), which might help modulate and balance the angiogenic response to prevent aberrant or excessive vessel formation [47–49]. The net effect of AMT in chronic wounds is moderated but enhanced angiogenesis, sufficient to relieve hypoxia and support granulation tissue without promoting pathological neovascularization.

#### Anti-Inflammatory and Anti-fibrotic Effects

Chronic wounds are locked in a state of persistent inflammation, which leads to extracellular matrix degradation and prevents progression to the proliferative phase of healing. AM traps and induces apoptosis in neutrophils and pro-inflammatory macrophages that come into contact with its stromal side [50]. It also skews macrophage differentiation towards the M2 (pro-healing) phenotype and away from the M1 (pro-inflammatory) phenotype [21]. In chronic venous ulcers treated with AMT, biopsies have shown reduced levels of inflammatory cytokines and fewer inflammatory cells compared to control wounds [37]. Concurrently, AMT’s anti-fibrotic action – via suppression of TGF-β signalling in fibroblasts – helps to prevent excessive scar tissue or fibrosis that can sometimes form around long-standing ulcers [23]. By reducing inflammation, AMT breaks the cycle of chronicity in wounds, allowing the healing process to move forward.

#### Antimicrobial Environment

An often underappreciated aspect of AMT in chronic wounds is its contribution to controlling infection. The AM contains natural antimicrobial peptides (such as beta-defensins, elafin, SLPI) and has been shown to exhibit antibacterial and even antiviral properties [51]. While AMT is not a replacement for systemic antibiotics when infection is present, its use can reduce bioburden. Some studies indicate that chronic ulcers treated with AM have lower bacterial counts and reduced incidence of infection compared to standard of care. The mechanism may involve both physical and biochemical factors: the amnion acts as a barrier against external contaminants and simultaneously releases antimicrobial factors locally [51]. In one report, amniotic membrane dressings in diabetic foot ulcers led to clearance of methicillin-resistant *Staphylococcus aureus* (MRSA) colonization without additional antibiotics, suggesting inherent antimicrobial activity [52]. This protective effect is highly beneficial, as chronic wounds are prone to infection which further delays healing.

In summary, AMT addresses multiple pathological aspects of chronic wounds. It supplies the chronic wound with what it is missing – a scaffold for cell migration, growth factors for cell division, signals for angiogenesis – and removes or counteracts detrimental factors like proteases, inflammatory cytokines, and microbes. By converting a hostile chronic wound milieu into a more acute-like, healing-friendly environment, AMT often achieves closure of wounds that were otherwise recalcitrant.

## Amniotic Membrane Transplantation in Tissue Regeneration

Beyond cutaneous wound healing, amniotic membrane transplantation has broader applications in regenerative medicine for various tissues and organs. Its ability to reduce fibrosis and support cell growth has been exploited in scenarios like tendon repair, cartilage regeneration, and even internal organ healing. The various applications and results of Amniotic Membrane Transplantation in Tissue Regeneration studies have been summarized in Table 1.

**Table 1:** Amniotic membrane transplantation applications and results in tissue regeneration studies

Application	Study	Type of wound	Subjects	Preparation method	Results
Burns	Tanideh et al. [53]	Second degree wound healing	72 adult male rats (in vivo)	Fresh HAM^1^	Significantly better performance in angiogenesis, granulation tissue formation and epithelization when compared to burned wool
	Momeni et al. [54]	Superficial burns	Rats (in vivo)	Cryopreserved Amniotic membrane extract Gel (AME-Gel)	Faster epidermal and dermal regeneration
	Ahuja et al. [30]	Small to moderate size burns	30 children (pediatric burns)	Dehydrated human amnion chorion membrane (dHACM)	Significantly faster rate of healing when compared to split thickness skin grafts
	Kakabadze et al. [55]	Non-healing radiation burn wounds	Inbred rats (in vivo)	Decellularized AM^2^	Increased the wound healing rate by 2.5 times
	Salehi et al. [56]	Second and third-degree burns	42 adult patients	Dried AM	Significantly faster wound healing as compared to Vaseline-impregnated gauze dressing
Tendon healing	De Castro Nicodemo et al. [57]	Achilli’s tendon injury	60 rats (in vivo)	Fresh AM	Reduced inflammatory response, better organization of collagen fibers and intense proliferation of fibroblasts during the tendon healing process
	De Girolamo et al. [58]	Collagenase induced tendinopathy	Sprague Dawley rats	Human Amniotic suspension allograft (ASA)	Improved fibre alignment and reduced cell density in the degenerated tendons in the treatment group
Ulcers	López Martinez et al. [59]	Calciphylaxis (chronic non-responsive ulcers)	Single adult patient	Cryopreserved AM	Enhanced epithelization and healing and reduced pain
	Lianju Qin et al. [60]	Calciphylaxis	Single adult patient	Human amnion-derived mesenchymal stem cells (hAMSCs)	Therapy shown to inhibit vascular calcification, stimulate angiogenesis and myogenesis, enhance re-epithelization
	Castiglia et al. [61]	Junctional epidermolysis bullosa (JEB) (corneal abrasions)	Single adult patient	HAM eye drops	Restoration of keratinocyte adhesions and induction of long-lasting remission
	Glat et al. [62]	Recalcitrant diabetic foot ulcers	60 adult patients	Dehydrated human amnion and chorion allograft (dHACA)	Healed significantly higher number of subjects at a significantly lower cost as compared to traditional tissue-engineered skin substitutes
Tissue engineering	Rita Singh et al. [63]	Split skin graft donor site	30 adult patients	Gamma-irradiated AM	Significant increase in average per day rate of epithelization and decreased pain
	Farzaneh Chehel et al. [64]	Random skin flaps (RSP)	Rats (in vivo)	Acellular amniotic membrane (AAM)	Therapy shown to improve the total number of mast cells and growth of capillaries in RSFs
	Ozkaya et al. [65]	Ischemic skin flaps	32 Male Rats (in vivo)	Fresh HAM	Significantly improved survival rate of skin flaps with increased blood vessels and decreased neutrophils on histology
Wound Healing	Barbosa Dias Campelo et al. [66]	Induced surgical injury	36 Rats (in vivo)	HAM	Significantly reduced inflammatory infiltrate and increased fibroblast concentration during the healing phase
	Murphy et al. [67]	Porcine skin (in vivo)	Full thickness wound models	Lyophilized amnion membrane powder, Amnion hydrogel	Significantly rapid wound healing driven by new epithelization and formation of mature epidermis and dermis
	Kim et al. [68]	Porcine skin (in vivo)	Full thickness surgical wounds on the back	AM- collagen dermal substitute	Faster healing rate and higher concentration of newly formed collagen as compared to other dermal substitutes
	Zhou et al. [69]	Mice models (in vivo)	Full thickness skin defects	Dried human acellular amniotic membrane (hAAM)	hAAM accelerated the formation of hair follicles and interfollicular dermis in subject mice
	Xue et al. [70]	92 adult patients	Reconstruction of the lower third of the nose	Freeze dried human acellular amniotic membrane (hAAM)	Significantly reduced hemostasis time and pain and improved wound healing as compared to Vaseline gauze treatment group
	Farzamfar et al. [71]	Rats (in vivo)	Full thickness excisional skin wound model	Decellularized human amniotic membrane (DAM)	Macroscopically – increased rate of wound closure and better cosmetic appearance Microscopically—higher collagen fiber synthesis and maturation

^1^*HAM* Human Amniotic Membrane

^2^*AM* Amniotic Membrane

### Tendon and Musculoskeletal Healing

Tendons and ligaments are prone to injury and typically heal slowly, often with scar formation that impairs function. Researchers have explored wrapping injured tendons with AM to improve healing quality. In a rat model of Achilles tendon injury, the application of fresh human AM wrap led to a reduced inflammatory response early in healing and more organized collagen fibre deposition later, with a higher density of proliferating fibroblasts in the repair tissue [57]. Treated tendons demonstrated stronger mechanical properties and fewer adhesions than untreated controls, indicating that AMT helped regenerate tendon tissue in a more functional, scar-less manner [57]. In another study, an injectable amniotic suspension allograft improved collagen alignment and decreased cellular hyperplasia in a collagenase-induced tendinopathy model [58]. These outcomes are attributed to AM’s anti-scarring effect (limiting peritendinous fibrosis) and its provision of growth factors that guide the remodelling of tendon fibres. The successes in tendon healing hint at AM’s general utility in musculoskeletal regeneration: it can modulate the healing response to favour regeneration over repair, which is a central goal in regenerative medicine.

### Nerve Regeneration

One of the most exciting frontiers for AMT is in nerve tissue repair. Nerves in the peripheral nervous system (PNS) can regenerate to some extent, but recovery is often incomplete due to scarring and misaligned regrowth. Central nervous system (CNS) neurons have even more limited regenerative capacity. The anti-inflammatory and neurotrophic milieu provided by the amniotic membrane has shown potential to improve outcomes in both peripheral nerve injuries and spinal cord injuries.

#### Peripheral Nerve Repair

Peripheral nerve injuries such as transected or crushed nerves in the limbs, are commonly treated with microsurgical techniques like epineurial repair or nerve grafts [72]. Even with optimal repair, scar tissue can form around the injury site, and aberrant fibrosis may impede axon regrowth or cause painful neuromas [73]. Wrapping the injured nerve with an amniotic membrane has been proposed as a method to reduce perineural scarring and provide a conduit for axonal regeneration.

The amnion acts as a biocompatible nerve wrap that isolates the repair site from surrounding inflammatory tissues and releases factors that support nerve healing. Gärtner et al. observed improved functional recovery in a rat model of sciatic nerve repair when an AM wrap was used: at 12 months post-injury, electrophysiological assessments showed better nerve conduction in AM-treated nerves compared to controls [74]. In their study, 42 rats with sciatic nerve crush injuries were treated either with standard care or with AM plus corticosteroid (betamethasone); the AM + steroid group had superior nerve function outcomes at 8 weeks, highlighting the membrane’s synergistic effect with anti-inflammatory therapy [74]. A separate study with 72 rats demonstrated that AM wraps significantly reduced adhesion and scar tissue around repaired sciatic nerves over 4–12 weeks. Less scar means that regenerating axons can cross the injury site more freely and reconnect with their targets [75].

Mechanistically, the amniotic membrane’s contribution to nerve healing is linked to its cell components and secreted factors. The membrane contains Schwann cell–like effects in that it produces growth factors such as nerve growth factor (NGF), brain-derived neurotrophic factor (BDNF), and glial cell line-derived neurotrophic factor (GDNF) in small amounts [76]. It also provides a basal lamina-like substrate for axons to grow along. Importantly, the amnion limits fibrosis at the nerve repair site, which is critical because fibrotic tissue can physically block axonal extension and create a diffusion barrier for nutrients [77, 78].

In practical application, surgeons have used both intact AM wraps and processed AM conduits for peripheral nerves. A two-layer AM wrap (with the epithelial side facing outward to avoid adhesions and the stromal side against the nerve) has been shown to improve outcomes in rat peroneal nerve transections: the AM wrap group had faster return of motor function (measured by toe spread reflex) and better histological nerve continuity than unwrapped repairs [76]. Compound muscle action potentials and nerve conduction velocities were closer to normal in the AM group, and the target muscles (e.g., tibialis anterior) showed improved structural preservation [76]. These findings underscore that AM not only acts as a passive barrier but actively enhances the intrinsic regenerative processes of peripheral nerves.

Clinical data, though still limited, are promising. Case series of patients with nerve compression syndromes (like carpal tunnel or peroneal nerve entrapment) treated with AM wraps during surgical decompression have reported reduced postoperative fibrosis and quicker symptom relief [79]. Some peripheral nerve graft products now incorporate AM as a surface coating to combine the benefits of guidance conduits with anti-scarring protection. Overall, amniotic membrane use in peripheral nerve injuries appears to promote a more favorable healing environment: one of reduced inflammation, limited fibrosis, and enriched with growth cues for axonal regrowth**.** These attributes can translate to improved functional recovery, which is the ultimate goal in nerve repair.

#### Central Nervous System (Spinal Cord and Brain) Applications

Unlike peripheral nerves, the central nervous system does not spontaneously regenerate effectively after injury. Spinal cord injuries (SCI) in particular lead to permanent neurological deficits largely because of the formation of glial scars and an inhibitory milieu for neuron regrowth. In this context, AMT has been investigated as a means to modulate the post-injury environment in the spine.

A systematic review by Moore et al. on amniotic membrane use for various spinal pathologies found that amnion-derived products significantly reduce epidural fibrosis after spinal surgeries, such as laminectomy or discectomy [80]. Epidural fibrosis can cause nerve tethering and chronic pain; thus, reducing it is beneficial. The review also noted improved spinal fusion rates and better postoperative pain outcomes when AM was used as an adjunct in spinal fusion surgeries [80].

In a similar study using spinal cord injury models, Ting-gang Wang et al. tried a tissue-engineering approach: they seeded human amniotic epithelial cells onto a silk fibroin scaffold and implanted this into a rat’s injured spinal cord. The result was a significantly smaller glial scar and a reduction in inflammatory cells at the injury site, alongside improved locomotor function in treated rats compared to those with scaffold alone [81].

In another line of investigation, human AM itself (as a patch) has been laid over spinal cord lesions in animal models [82]. These studies showed that AM can survive for some time in the spinal injury environment and possibly serve as a substratum for native neural cells to migrate into the injury site. Although true neural regeneration in the CNS remains extremely challenging, AMT appears to mitigate secondary injury processes (like glial scar formation) that usually limit any chance of regeneration.

The use of amniotic membrane for dural repair in neurosurgery is another emerging area. The dura mater is the outer membrane covering the brain and spinal cord, and it is often breached in surgeries or by trauma. An ideal dural substitute should be watertight (to contain cerebrospinal fluid), non-immunogenic, and promote tissue integration. A review of HAM for duraplasty found that it performs comparably to synthetic dural substitutes in terms of preventing CSF leaks and infections, with the advantage of being a natural collagenous membrane [83]. However, there were a few cases of CSF leaks reported, indicating that more robust fixation or multi-layer usage of AM may be necessary for it to serve as a dependable dural patch. Choi et al. studied rats undergoing laminectomy and noted that irradiated freeze-dried AM placed over the laminectomy site significantly reduced epidural scar formation and inflammatory infiltration compared to controls [84]. This has implications for human spinal surgeries as using AM could lower the incidence of postoperative adhesion-related complications, such as nerve root impingement.

In the brain, clinical experience with AMT is limited but expanding. There are case reports of AM being used in skull base surgery to repair defects in the membrane separating the brain from the nasal sinuses, successfully preventing CSF leaks [85, 86]. Additionally, the neuroprotective factors in AM (like endogenous antioxidants, anti-apoptotic factors) are of interest for stroke or traumatic brain injury, though so far these are theoretical or in early experimental stages.

While CNS regeneration is far from achieved, amniotic membrane applications in neurosurgery and spinal repair focus on protection and support: reducing scarring, preventing adhesions, and potentially providing a bridge for cells to facilitate healing. AMT creates a permissive environment – whether around a peripheral nerve or in a laminectomy site – that can prevent complications and possibly enhance the body’s attempt to repair nerve tissues.

## Amniotic Membrane Transplantation in Ophthalmology

Ophthalmology is the field where modern AMT was re-established and where it remains a cornerstone of treatment for various ocular surface disorders. The eye’s surface (cornea and conjunctiva) and adnexa are particularly well-suited to amniotic membrane applications because they are accessible, and the membrane’s transparent, thin, and pliable nature is ideal for ocular use. Over the past few decades, AMT has been broadly adopted for indications such as persistent corneal epithelial defects, corneal ulcers, chemical burns, pterygium excision, conjunctival surface reconstruction, and as support for limbal stem cell transplantation.

### Cornea and Ocular Surface

In ocular surface reconstruction, AMT can be applied either as a graft (inlay technique**)**, where the membrane is placed into a tissue defect (for example, filling a corneal ulcer or stromal thinning), or as a patch (overlay technique), where it is laid on top of the surface as a biological bandage [87]. It can also be used in a layered fashion (fill-in) for deeper corneal or scleral defects, with multiple membranes stacked to achieve the desired thickness [87]. The choice of technique depends on the pathology: for a corneal perforation or deep ulcer, a multilayer inlay AMT might be used to restore integrity, whereas for a non-healing epithelial defect, a single-layer patch can promote healing [87].

In cases of persistent epithelial defects and trophic corneal ulcers (often seen in neurotrophic keratitis or post-infectious keratitis), AMT provides a basement membrane substitute on which corneal epithelium can grow. It also delivers the heavy-chain hyaluronic acid/Pentraxin 3 complex (HC-HA/PTX3) from the AM stroma that has anti-scarring and anti-inflammatory effects, helping the cornea heal with less haze [88, 89].

A prospective study by Badotti Linhares et al. on refractory corneal ulcers showed that cryopreserved AMT led to epithelial healing in the majority of cases and reduced stromal inflammation, preventing progression to perforation [90]. In chemical or thermal burns of the eye, AMT applied early can reduce acute inflammation and pain and serve as a temporary ocular surface while native cells recover. As noted earlier, AMT in acute burns promotes epithelialization and can sometimes obviate the need for emergent keratoplasty.

For corneal perforations or descemetoceles (deep ulcers where only Descemet’s membrane remains), multilayered AM grafts have been used to seal the defect and stabilize the eye [91].

He et al. reported a case of chronic graft-versus-host disease with a corneal descemetocele that was successfully managed with a multilayer AMT, achieving symptom relief and structural stability of the globe until a definitive corneal transplant could be performed [91].

For pterygium surgery, where an abnormal conjunctival tissue encroaches onto the cornea, AMT is an established alternative to conjunctival autografts. When a pterygium is removed, placing an AM graft on the sclera can reduce recurrence rates by providing a healthy substrate and suppressing fibrosis and neovascularization associated with the regrowth of the pterygium [92]. With proper technique (often combined with anti-metabolites like mitomycin C or intraoperative steroids), pterygium recurrence with AMT is comparable to that with conjunctival autografts in many studies, with the cosmetic outcome being very satisfactory – the AM integrates and the ocular surface regains a normal appearance [93].

Conjunctival surface reconstruction using AM is also common after the removal of tumors (like conjunctival melanomas or extensive squamous neoplasia) or in cicatricial diseases (e.g., Stevens-Johnson syndrome, ocular cicatricial pemphigoid) [94]. The AM, rich in collagen and laminin, supports the regrowth of conjunctival epithelium and helps recreate a smooth, wettable surface, while its anti-inflammatory properties limit fibrosis that could restrict eye movements or cause eyelid adhesions [94].

### Limbal Stem Cell Deficiency (LSCD)

In conditions where the limbal stem cells of the cornea are deficient (due to burns, Stevens-Johnson syndrome, or aniridia, for example), AMT is used as part of the strategy to restore the corneal surface [95, 96]. Sometimes it is placed as a temporary measure to suppress inflammation before limbal stem cell transplantation. Other times, cultured stem cells are expanded on an AM carrier (since the AM basement membrane is an excellent substrate for epithelial cell growth) and then transplanted to the patient – a technique known as Cultivated Limbal Epithelial Transplantation (CLET) [97]. The AM in these cases serves as a scaffold delivering the new stem cells to the ocular surface. Even Simple Limbal Epithelial Transplantation (SLET), which involves small pieces of limbal tissue from a donor eye, often utilizes AM either during surgery or as a bandage after, to facilitate the spread of new epithelial cells [97]. In chronic LSCD, AMT alone (without stem cell transplant) won't permanently restore vision, but it can significantly improve comfort, reduce conjunctivalization of the cornea, and prepare the eye for further procedures.

### Other Ocular Applications

Beyond the cornea and conjunctiva, the amniotic membrane has found novel uses in other ophthalmic surgeries. In strabismus surgery, which involves extraocular muscle operations to correct misalignment of the eyes, scar tissue formation can sometimes lead to complications like restricted eye movements or the need for re-operations. AMT has been applied to the sclera or muscle insertion site during strabismus surgery to act as a spacer or buffer to reduce postoperative adhesions. Kassem and El-Mofty (2019) reported that using AM in strabismus surgery helped prevent muscle reattachment to the sclera in undesirable ways and reduced fibrosis, thereby improving the success of reoperations [98]. Tugcu et al. (2013) similarly found that AMT in strabismus reoperations led to better ocular motility outcomes compared to controls, as the membrane minimized scar tethering between the muscle and orbit [99].

Another innovative application is in vitreoretinal surgery. Yamada et al. (2001) treated a difficult case of fat adherence syndrome which is a complication of retinal detachment surgery where orbital fat scars onto the scleral buckle, by placing an amniotic membrane to separate the fat from the ocular surface; this helped resolve the adhesion and improve ocular movement [100].

In recent years, retina surgeons have explored using AM as an intraocular patch. Caporossi et al. (2019) have used human AM as a plug to successfully close refractory macular holes, particularly in cases of high myopia where conventional surgery fails [101]. The thin AM is placed in the hole in the macula to serve as a scaffold for the ingrowth of retinal cells or glia, promoting anatomical closure of the hole and thereby improving or stabilizing vision [102]. Initial results from such cases are promising, indicating anatomic success in closing chronic, large macular holes that otherwise would remain open.

Similarly, AMT has been attempted to repair retinal detachments associated with giant tears: the AM can tamp down the retina and act as a biologic tissue patch to close retinal breaks. While these uses are still experimental and not yet mainstream, they exemplify the expanding frontiers of AMT in ophthalmology.

### Techniques and Integration

Depending on the ophthalmic application, AM can be used fresh, cryopreserved, or dried. Cryopreserved AM (available in products like ProKera® which is a ring-mounted AM insert) retains many of the native factors and is often preferred for its better biological activity [103]. In surgery, AM is typically sutured or glued onto the eye. A sutureless option in ophthalmology is the ProKera device, where a piece of AM is held by a ring that sits on the eye like a large contact lens; it has FDA approval since 2003 for use in corneal diseases and allows easier application in the clinic without surgical sutures [103].

In summary, AMT in ophthalmology has become a standard of care for many surface diseases. It capitalizes on the membrane’s ability to promote a healthy, anti-inflammatory healing environment on the delicate ocular tissues, which demand clarity and minimal scarring for optimal function. The versatility of AMT in the eye – from surface reconstruction to acting as an internal patch – showcases the membrane’s adaptability. As new techniques emerge (like combining AM with stem cells or using it in posterior segment surgeries), the role of AMT in ophthalmology continues to expand. The multiple applications of Amniotic Membrane Transplantation in Ophthalmology have been summarized in Table 2.

**Table 2:** Applications and results of amniotic membrane transplantation in ophthalmology

Application	Study	Subjects	HAM^1^ preparation	Results
Corneal Pathologies				
Corneal Perforation	George Hayek et al. [104]	Case Report (case of herpetic keratitis)	Lyophilized amniotic membrane graft	Rapid visual recovery, the corneal defect was closed and the globe integrity was preserved without the need for keratoplasty
	Ya-Li Du et al. [105]	Case series (16 cases of fungal keratitis)	Multilayer AMT^2^ in conjunction with bipedicle conjunctival flap	Improvement of visual acuity, preservation of globe integrity and no recurrence of FK in the cases
Corneal Descemetocele	Yunjiao He et al. [91]	Case of chronic ocular graft-versus-host disease (oGVHD)	Multilayer AMT	Resolution of clinical symptoms, restoration of the ocular surface structure, and helped buy time as the patient awaited corneal transplantation
Corneal Ulcers	Anna Carolina Badotti Linhares et al. [90]	Prospective study (11 cases of refractory neurotropic ulcers)	Cryopreserved AMT	Complete epithelial closure (60% of cases), Complete resolution of pain (66% of cases)
	H J Chen et al. [106]	Case series (16 eyes of 15 cases with severe neurotropic ulcers)	3-layer AMT	Rapid epithelization in 76.4% of cases
	J S Kim et al. [107]	Prospective study (21 cases of infectious corneal ulcer)	Anti-infective (antibacterial/antiviral/antifungal) soaked AMT	Successful healing of corneal surface and no recurrence of microbial infection
Band Keratopathy	Young Sam Kwon et al. [108]	Case series (2 cases of calcified band keratopathy)	Temporary amniotic membrane patch	Ocular surface was restored without pain or inflammation, no recurrence was observed
	D F Anderson et al. [109]	Case series (16 eyes from 15 cases)	AMT after primary surgical management	93.7% cases achieved complete epithelization, resolution of pain in all cases
Scleral Pathologies				
Scleromalacia	Jung Hwan Oh et al. [110]	Prospective study (8 cases)	AMT over the glycerin-preserved scleral graft	Marked improvement in visual acuity, rapid epithelization, loss of ocular pain and inflammation
Scleral ulcer	David Hui-Kang Ma et al. [111]	Case series (4 cases of infectious scleral ulcers with persistent scleral melting)	Cryopreserved HAM^2^	Decreased melting and inflammation at lesion site, complete re-epithelization of the sclera
Necrotizing scleritis	Shreya Thatte and Lalita Gupta [112]	Case of surgically induced necrotizing scleritis (SINS)	Multilayered AMT	Improvement of scleral melt, complete resolution within 15 days of AMT, no recurrence
Scleral perforation	M T Rodriguez-Ares [113]	Case of Marfan syndrome with scleral perforation	AMT along with scleral homograft	Complete restoration of perforation with no signs of inflammation or infection
Conjunctival Pathologies				
Symblepharon	Raina Goyal et al. [114]	Retrospective case review (5 eyes)	AMT after primary surgical management	Restoration of conjunctival surface, improved visual acuity (66.67%)
Pterygium	Asadollah Katbaab et al. [93]	Interventional case series (50 eyes from 50 patients)	AMT after excision	Resolution of conjunctiva, 2% recurrence rate
Conjunctivochalasis	D Meller et al. [115]	Prospective study (47 eyes from 40 patients with refractory conjunctivochalasis	AMT	Improvement in symptoms, resolution of episodic epiphora (83.3%) and restoration of smooth, quiet and stable conjunctival surface
	Ahmad Kheirkhah et al. [116]	Case series (25 eyes of 16 patients)	Sutureless AMT with fibrin glue	Smooth conjunctival surface and symptomatic improvement
Other pathologies				
Glaucoma Surgery (Repair of late onset leaking glaucoma filtering blebs)	D L Budenz et al. [117]	Prospective RCT^3^	AMT	AMT is not an effective alternative to conjunctival advancement

^1^*HAM* Human Amniotic Membrane

^2^*AMT* Amniotic Membrane Transplantation

^3^*RCT* Randomized Control Trial

## Challenges and Considerations

While AMT offers numerous benefits, there are important challenges and considerations that temper its use and development. These include regulatory issues, ethical considerations of tissue sourcing, and practical limitations such as variability between amniotic grafts.

### Regulatory Approval and Clinical Use

Amniotic membrane products must meet strict regulatory standards to ensure safety and efficacy. In the United States, the FDA regulates human tissue products under 21 CFR Part 1271, which covers the screening of donors, processing, and labeling of tissue-based products [118]. Several AMT products have achieved FDA clearance or approval. For example, ProKera® (Bio-Tissue Inc.) – a cryopreserved AM clipped into a self-retaining ring – was approved in 2003 for ophthalmic use and introduced clinically in 2020 as a sutureless device for corneal healing [103]. This device simplifies ocular AMT by allowing in-office placement under topical anesthesia, delivering the amniotic membrane directly to the cornea without surgery. In wound care, EpiFix® (MiMedx Group) is a dehydrated amnion/chorion membrane allograft that is widely used for chronic ulcers; it is processed from consented placental donations and is regulated by the FDA as a wound healing graft [119]. Similarly, NEOX® (Amniox Medical) is a cryopreserved composite of amniotic membrane and umbilical cord, employed for treating difficult dermal ulcers and known for its rich content of regenerative factors [120]. AmnioExcel® (Integra LifeSciences/Dermasciences) is another dehydrated AM product for ulcers that has been processed to preserve extracellular matrix components and growth factors [121]. These commercial products have made AMT more accessible, but they also introduce considerations of cost and standardization.

One challenge is the batch-to-batch variability inherent in biologic tissues. Amniotic membranes from different donors (or processed in different ways) may not be identical in terms of thickness, growth factor levels, or cell viability [122]. This can lead to inconsistencies in clinical outcomes. Manufacturers attempt to standardize products by specifying inclusion criteria for donors (e.g., full-term cesarean deliveries from healthy mothers), and by using quality-controlled processing (e.g., specific cryopreservation protocols, sterilization steps) [123]. Despite this, clinicians sometimes observe that one graft “takes” or works better than another.

Another regulatory aspect is classification: Many amniotic products are marketed under the framework of “361 HCT/Ps”(Human Cells, Tissues, and Cellular and Tissue-Based Products) which do not require pre-market approval if they are minimally manipulated and intended for homologous use [124]. However, if an AM product is more than minimally manipulated or claimed for non-homologous use (for example, as a treatment for osteoarthritis pain), it may be regulated as a drug or device, necessitating clinical trials. The line can be blurry, and regulators worldwide are refining policies as the field evolves. The World Health Organization and various national health agencies have guidelines to ensure safety – including donor screening for infectious diseases (to prevent transmission of HIV, hepatitis, etc.), traceability of grafts, and proper informed consent for tissue donation.

### Ethical and Logistical Considerations

The ethical sourcing of amniotic membranes is generally straightforward, as it is obtained from placentas that would otherwise be discarded after childbirth. Donors (mothers) must give informed consent for the use of their placental tissues, and they are screened to confirm the absence of infectious diseases. Only the mother’s consent is required as the fetus/infant is not harmed and the tissue is considered part of the maternal delivery process [125]. Donors have the right to withdraw consent at any time before the tissue is used, and this autonomy is protected by law [125].

In many jurisdictions, including the EU and US, there are strict rules to ensure anonymity and no financial coercion: the identity of donors is kept confidential (linked only through tissue banks if needed for tracking) and the sale of human tissues is prohibited [126]. Companies can charge for processing and handling, but not for the tissue itself [127]. The non-profit tissue bank model or tightly regulated commercial model is employed to maintain public trust [127].

There is a general consensus that using birth tissue (amnion) for therapy is ethically acceptable and even beneficial, as it repurposes biological material to save lives and heal patients. However, some ethical questions occasionally arise: for instance, are all donors fully informed that their placenta may be used in products sold for profit? Transparency in the consent process is vital.

Another consideration is the concept of microchimerism and cell persistence. When living amniotic cells are introduced into a patient, there is a small possibility that some of these cells could integrate into the host tissues and persist long-term [128]. This phenomenon, akin to fetal-maternal microchimerism during pregnancy, raises questions about long-term effects [129, 130]. Might these cells differentiate or influence immune responses far in the future? Thus far, no adverse consequences have been clearly linked to microchimerism from AMT; in fact, microchimeric fetal stem cells are hypothesized to aid in tissue repair rather than cause harm [131]. Nonetheless, long-term follow-up of AMT recipients, especially those who receive cellular amnion products (like amniotic suspension injections containing live cells), is advisable to monitor for any unexpected outcomes.

Finally, there are cost and accessibility issues. Amniotic membrane products can be expensive, and insurance coverage varies. Ensuring that this therapy is available to patients who need it (for instance, those with diabetic foot ulcers at risk of amputation, or patients in low-resource settings with burns) remains a challenge. Researchers are exploring more cost-effective preservation techniques and even decellularized AM (which might have a longer shelf life and potentially lower cost) to broaden accessibility.

Despite these challenges, the trajectory of AMT is forward-moving. Regulatory bodies have increasingly recognized AMT as a valuable option, and standards are being refined rather than AMT being hindered. Ethically, it stands as a positive example of using donated biological materials to heal others. The ongoing challenge is to maintain rigorous standards and continue scientific evaluation so that AMT therapies are safe, effective, and optimized.

## Emerging Applications

The applications of human amniotic membrane are continually expanding as research unveils new ways to harness its properties. Initially concentrated in dermatologic wounds and ocular surface disease, AMT is now making inroads into regenerative medicine, tissue engineering, and even systemic therapies.

### Advanced Regenerative Medicine and Engineering

One trend is the integration of amniotic membrane with other cutting-edge therapies. For example, combining AMT with stem cell therapy has shown synergistic effects. Mesenchymal stem cells (MSCs) placed on or delivered with an amniotic membrane may exhibit enhanced survival and function. Studies indicate that co-transplanting AM with MSCs amplifies the immunomodulatory and regenerative effects of the stem cells, aiding in tissue repair in models of ocular injury and myocardial infarction [132, 133]. The AM provides a hospitable niche for stem cells, and in turn, the stem cells may secrete additional growth factors that work in concert with the amnion’s factors [132, 133]. In ophthalmology, researchers have developed amniotic membrane extract eye drops that can be used to support corneal epithelial healing and even limbal stem cell growth, offering a non-surgical AMT approach for conditions like limbal stem cell deficiency [134].

Another innovation is using amniotic membrane as a building material in bioengineering. A fascinating study by Li et al. created a decellularized amniotic membrane hydrogel for intrauterine adhesion (IUA) therapy. In a rat model, this AM hydrogel reduced scar formation in the uterus and improved endometrial regeneration, suggesting a potential application in fertility-preserving treatments for Asherman’s syndrome [135].

The breadth of AMT’s potential is also being explored in fields like otorhinolaryngology and gender-specific medicine. For example, after extensive head and neck cancer surgeries, AMT has been used to cover pharyngeal or oral cavity defects to aid mucosal regeneration (with some success in reducing healing time and improving mucosal quality). In a reported case, after resecting laryngeal chondrosarcoma, surgeons applied an AM patch in the airway reconstruction, which was noted to integrate well and support the regrowth of respiratory epithelium [136].

Likewise, Marton et al. used AM as a dural patch in neurosurgery for spina bifida cases (open myelomeningocele), and it proved effective as a reconstructive and anti-adhesive material, essentially acting as an artificial dura to protect the spinal cord [137]. In orthopaedic research, amnion yarn or threads have been fabricated from processed amniotic membranes to weave vascular grafts; these show adjustable mechanical properties and good biocompatibility, pointing to a future where tissue-engineered blood vessels could incorporate amniotic fibers [138]. Furthermore, a large retrospective series has reported significant improvements in knee osteoarthritis symptoms after injections of micronized dehydrated amnion/chorion (mdHACM) into joints, with quality-of-life scores rising for months post-treatment [139].

This suggests amniotic tissue might have disease-modifying effects in degenerative joint disease, possibly through its anti-inflammatory and cartilage-protective factors.

## Future Directions

As AMT continues to evolve, future research will likely explore innovative clinical applications, expanding its use into previously underexplored or entirely novel therapeutic areas.

For instance, nanotechnology and AMT could go hand in hand. Researchers are exploring loading amniotic membranes with nanoparticles to deliver drugs or enhance antioxidant capacity. One exciting avenue is the use of nanoceria (cerium oxide nanoparticles), which are potent catalytic antioxidants, in conjunction with AMT [140]. Nanoceria can repeatedly scavenge reactive oxygen species. In retinal degeneration models, nanoceria localized in the outer retina were shown to protect the retinal pigment epithelium by reducing oxidative stress and inflammation [141]. Translating this to AMT, a future therapy might involve embedding nanoceria into an AM patch applied to a wound or an ocular surface, thereby combining the membrane’s biological benefits with sustained antioxidative protection. This could be particularly useful in chronic wounds or ischemic tissues where oxidative damage impedes healing. Early research has indeed demonstrated that intravitreal nanoceria can decrease pathological neovascularization (by reducing VEGF levels) in eye disease models [142], hinting that an AMT-nanoceria hybrid could modulate the wound microenvironment in favor of regeneration over chronic inflammation.

In gynaecology, beyond intrauterine adhesion treatment, AMT is being evaluated for vaginal reconstructive surgeries(e.g., in Müllerian agenesis or gender-affirming surgeries) where a graft that can support epithelium and prevent scar contracture is needed. For instance, using AM in pelvic organ prolapse repairs or urethral reconstruction. Indeed, some urologic surgeons have begun employing AM wraps for penile prosthesis implants or urethroplasties to reduce fibrosis and improve outcomes in complex cases [143], bridging the gap between regenerative medicine and gender-specific surgical needs.

Vitamin D3 is a hormone known for its role in immune regulation and tissue repair. Vitamin D deficiency has been linked with poor wound healing and greater inflammation. Recent findings in ophthalmology suggest that adequate vitamin D3 can support corneal health and even mitigate AMD progression. Vitamin D3 downregulates pro-inflammatory cytokines (like IL-1α, IL-6, IL-8) in keratinocytes and upregulates antimicrobial peptides [144]. We foresee interest in optimizing patients’ vitamin D status or even topically applying vitamin D analogues alongside AMT to enhance healing outcomes. In practical terms, this could mean that in the future, chronic wound patients receiving AMT might also receive a vitamin D3 supplement regimen to synergistically reduce inflammation and promote re-epithelialization.

Targeting specific inflammatory pathways is another horizon. One such target, as mentioned earlier, is the P2X7 receptor involved in ATP-mediated inflammation. Inhibitors of P2X7 (like the natural compound dihydrotanshinone) have been shown to preserve tissue barriers (e.g., blood-retinal barrier) under stress by blunting the inflammatory cascade [25]. In the context of AMT, pharmacologically modulating P2X7 could amplify AM’s innate anti-inflammatory effects. For instance, applying an AM graft together with a P2X7 antagonist drug at a site of injury might result in even lower levels of IL-1β and TNF-α than AM alone, thereby fostering a more regenerative environment. While this is speculative, it represents how understanding molecular pathways can open up new combined therapies featuring AMT.

Perhaps one of the most futuristic angles is using amniotic membrane as a platform for gene delivery. The concept is that amniotic cells could be transduced with therapeutic genes (for instance, a gene encoding a growth factor or anti-inflammatory cytokine) and then delivered via the membrane to a patient. The biocompatibility, large surface area, and supportiveness of AM make it an intriguing scaffold for such gene therapy approaches [145]. In liver disease models, amniotic epithelial cells have been experimentally gene-modified and used to treat congenital metabolic disorders, showing promising results [145]. The idea could extend to wound healing – imagine an AM graft engineered to overexpress Nrf2 or HO-1, providing a localized boost in anti-oxidative defense for a non-healing ulcer, or an AM seeded with cells carrying genes for vascular endothelial growth factor (VEGF) to stimulate blood vessel growth in an ischemic limb wound. Because the AM is naturally adept at delivering cells and factors, it could serve as an ideal vehicle for gene-enhanced regenerative therapy [145]. This remains a nascent area of research, but given the rapid advancements in gene editing (like CRISPR) and cell therapy, it is a plausible future direction.

Future directions are not limited to new applications but also include optimizing the amniotic membrane product. Ongoing research is addressing how to create “off-the-shelf” AM that is more potent and easier to use. This includes lyophilization techniques that preserve more growth factors, composite grafts that combine amnion with other matrix materials (for added strength or delayed release of factors), and sprayable or injectable forms of amniotic membrane matrix for irregular wounds [146]. We might see an AM-derived topical gel or ointment that could be applied like a cream but still deliver key amniotic factors to a wound [147].

As we integrate new technologies (nanoparticles, gene editing, biofabrication) with this age-old biological material, we anticipate the development of hybrid solutions that could tackle some of the most stubborn challenges in tissue regeneration. The future of AMT lies in such multidisciplinary convergence – ensuring that this naturally derived therapy remains at the cutting edge of regenerative science.

## Conclusion

This review highlights the versatile applications and evolving landscape of amniotic membrane transplantation (AMT) in wound healing, tissue regeneration, and ophthalmology. We have seen that in acute wounds, AMT reliably reduces pain and infection risk while promoting faster epithelialization, acting as a biologically active dressing. In chronic wounds, it helps break the inflammatory cycle, encouraging re-epithelialization and angiogenesis and thereby facilitating closure of ulcers that otherwise resist healing. AMT also plays a critical role in nerve regeneration, showing potential in repairing peripheral nerves and even aiding spinal cord injury treatments by promoting neural recovery and reducing scar tissue formation. In ophthalmology, AMT is firmly established for corneal surface reconstruction, conjunctival repairs, and treating conditions like dry eye syndrome and limbal stem cell deficiency, with emerging uses in retinal surgeries and glaucoma underscoring its expanding utility. Despite its many benefits, we must acknowledge the challenges: clinical success can vary by condition; there are ethical and logistical considerations in donor tissue procurement; and regulatory hurdles must be navigated to ensure the safe and standardized use of AM products. These challenges are being addressed through improved processing standards and robust clinical trials.

Importantly, the field is moving forward. Future directions include exploring AMT in novel areas such as nerve repair (e.g., as adjuncts in neurosurgery for spinal injuries), orthopaedic applications (for cartilage/tendon repair and even joint preservation), and unique scenarios like airway or gynecologic reconstruction. We also discussed innovative combinations – from integrating nanotechnology and antioxidants to leveraging gene therapy and stem cell science – that could amplify AMT’s therapeutic impact. The phenomenon of microchimerism, wherein donor amniotic cells might persist in the recipient, opens intriguing questions about long-term regenerative interactions between graft and host. With increasing regulatory approvals and growing clinician familiarity, AMT continues to gain traction as a promising tool in regenerative medicine across multiple specialties. In conclusion, amniotic membrane transplantation stands as a unique convergence of biological complexity and clinical efficacy – a platform upon which future therapies will likely build to achieve even greater healing outcomes for patients.

## Data Availability

Not applicable.
